# Pennsylvania’s Law Vindicated

**Published:** 1901-08

**Authors:** J. W. Sallade

**Affiliations:** President Veterinary Medical Examining Board of Pennsylvania; Auburn, Pa.


### COMMUNICATION.

### PENNSYLVANIA'S LAW VINDICATED.

AUBURN, PA., July 3, 1901.

EDITOR JOURNAL OF COMPARATIVE MEDICINE, ETC.:

IT may, perhaps, be of interest to the profession to read that the Act of 1895 was vindicated in the courts of Schuylkill County.

For several years one Jabez Burkes has given our State Board considerable trouble. Repeated complaints were filed with the board, and each time Burkes was written to and asked to stop practising veterinary surgery. These tactics were pursued, and Mr. Burkes led the board a lively race while it lasted; but at last he came to grief, and is now serving out a sentence in the Schuylkill County Jail.

Mr. Jabez Burkes has somewhat new and novel methods. He styled himself a "veterinary nurse." He would find out where there were lame horses, then offer to treat, nurse, and cure the same for a stipulated sum. No cure no pay.

In a few days he would claim the animal sound and demand his money. If not forthcoming, threats of lawsuits, followed by a compromise, generally wound up the case; and after disturbing the peace and quiet of a community and ruffling the temper of the local vets and horseshoers—exit Mr. Burkes.

He was finally arrested at Mahanoy City on a warrant sworn out by the subscriber, where he had gone from Tamaqua after receiving notice to quit, in writing, from Dr. Hoskins, secretary of the board, and from myself personally. I went there to see the fellow, and he had the audacity and impudence to abuse me and the board, claiming that the law was unconstitutional and that we were persecuting him.

There being no other remedy, I swore out a warrant, charging him with illegally practising veterinary medicine and surgery. His arrest followed, and he was committed by the magistrate in default of three hundred dollars bail for trial by court and jury.

On July 19, 1901, the case was called before Judge Bechtel. The defendant was represented by able counsel, the board by Attorney N. Heblich. The suit was brought in the name of the board. Counsel for the defendant asked what we expected, and was told either a plea of guilty or trial.

After a brief consultation with his client, the prisoner was arraigned and asked to plead. He entered a plea of guilty, and promised the court never to violate the same statute again if it would deal leniently with him. This the Court said it would do, but reminded Burkes that should he ever again come before the Court on a similar charge he would deal severely with him.

The sentence of the Court was fifteen dollars fine and costs of prosecution, and stand committed until the sentence of the Court is complied with. The prisoner will, no doubt, serve three months in jail, and then avail himself of the benefits of the insolvent laws.

The profession may take heart; the law is vindicated. A successful prosecution of a violator is a matter of record, and stands as an object lesson to others.

Yours truly,

J. W. SALLADE, V.S.,

President Veterinary Medical Examining Board of Pennsylvania.

